# 1188. Efficacy of Copper Impregnated Self-Sanitizing Surface Against *Clostridioides difficile* Spores

**DOI:** 10.1093/ofid/ofac492.1023

**Published:** 2022-12-15

**Authors:** Landon Ashby, Thanuri Navarathna, John David Coppin, Piyali Chatterjee, Marjory D Williams, Morgan Bennett, Hosoon Choi, Munok Hwang, Jing Xu, Richard Nelson, Yonhui Allton, Chetan Jinadatha

**Affiliations:** Central Texas Veterans Health Care System, Temple, Texas; Central Texas Veterans Health Care System, Temple, Texas; Central Texas Veterans Health Care System, Temple, Texas; Central Texas Veterans Health Care System, Temple, Texas; CTVHCS, Temple, Texas; Central Texas Veterans Health Care System, Temple, Texas; Central Texas Veterans Health Care System, Temple, Texas; Central Texas Veterans Health Care System, Temple, Texas; Central Texas Veteran Health Care System, Temple, Texas; Salt Lake VA, Salt Lake City, Utah; Central Texas Veterans Healthcare System/Department of Veterans Affairs, Temple, Texas; Central Texas Veterans Health Care System, Temple, Texas

## Abstract

**Background:**

*Clostridioides difficile* (*C. diff*) is one of the most common causes of healthcare-associated infections (HAIs), leading to increased hospital stay, cost, and mortality. Elimination of *C. diff* spores is difficult as they are resistant to kill by common hospital grade disinfectants. Copper impregnated self-sanitizing surfaces (SSSCu) provide continuous reduction of vegetative pathogens, potentially lowering the risk of infections, but their efficacy on *C. diff* spores has not been previously evaluated.

**Methods:**

Control (no copper) and copper coupons containing 20% copper-oxide were inoculated with varying *C. diff* spore loads ranging from 10^5^ to 10^7^ spores, prepared using environmental protection agency protocol, with or without 5% fetal bovine serum (FBS) soil load. After 4 hours of contact time, the *C. diff* spores were recovered and tested for viability. The efficacy of copper (log_10_ kill) was estimated using a Bayesian linear multilevel model.

**Results:**

After 4 hours, the copper coupons, at mean initial spore load (6.67 log_10_) and no soil load, had a mean 1.21 (95% uncertainty interval: 1.13 - 1.31) log_10_ reduction compared to control coupons. With soil load, the treatment effect of copper decreased by 0.50 (95% uncertainty interval: 0.37 - 0.64) log_10_. Also, for each additional standard deviation (SD) increase in initial spore load there was a 0.59 (95% uncertainty interval: 0.47 - 0.72) log_10_ decrease in the treatment effect of copper. The soil load in combination with increasing initial spore load further decreased the treatment effect of copper by an additional 0.19 (95% uncertainty interval: 0.01 - 0.38) log_10_ for each SD increase in initial spore load.

**Conclusion:**

Copper coupons can substantially reduce *C. diff* spores after 4 hours by 50%-97% depending on the initial spore concentration and presence or absence of organic material. Higher initial spore loads or excess organic material may prevent the spores from coming in contact with the copper-impregnated surface thus decreasing the kill efficacy. Continuous sporicidal effect of copper-impregnated surfaces might prevent transmission of spores and help reduce HAIs. Further studies are needed to evaluate the efficacy on *C. diff* HAI rates.

**Disclosures:**

**Piyali Chatterjee, PhD**, AHRQ Grant # 1R03HS027667-01: Grant/Research Support|AHRQ Grant # 1R03HS027667-01: Central Texas Veterans Health Care System **Chetan Jinadatha, MD, MPH**, AHRQ R01 Grant-5R01HS025598: Grant/Research Support|EOS Surfaces: Copper Coupons and materials for testing.

